# Functional Biological Activity of Sorafenib as a Tumor-Treating Field Sensitizer for Glioblastoma Therapy

**DOI:** 10.3390/ijms19113684

**Published:** 2018-11-21

**Authors:** Yunhui Jo, Eun Ho Kim, Sei Sai, Jin Su Kim, Jae-Min Cho, Hyeongi Kim, Jeong-Hwa Baek, Jeong-Yub Kim, Sang-Gu Hwang, Myonggeun Yoon

**Affiliations:** 1 Division of Radiation Biomedical Research, Korea Institute of Radiological and Medical Sciences, Seoul 01812, Korea; unyjjo@gmail.com (Y.J.); eunhokim8@gmail.com (E.H.K.); chojaemin09@naver.com (J.-M.C.); jihan918@kirams.re.kr (J.-H.B.); wjdduql@hanmail.net (J.-Y.K.); 2 Department of Bio-Convergence Engineering, Korea University, Seoul 02842, Korea; 3 Department of Basic Medical Sciences for Radiation Damages, National Institute of Radiological Sciences, Chiba 263-0024, Japan; sai.sei@qst.go.jp; 4 Division of RI-Convergence Research, Korea Institute of Radiological and Medical Sciences, Seoul 01812, Korea; kjs@kirams.re.kr (J.S.K.); hyeongi@kirams.re.kr (H.K.)

**Keywords:** tumor-treating fields, glioblastoma, sorafenib

## Abstract

Glioblastoma, the most common primary brain tumor in adults, is an incurable malignancy with poor short-term survival and is typically treated with radiotherapy along with temozolomide. While the development of tumor-treating fields (TTFields), electric fields with alternating low and intermediate intensity has facilitated glioblastoma treatment, clinical outcomes of TTFields are reportedly inconsistent. However, combinatorial administration of chemotherapy with TTFields has proven effective for glioblastoma patients. Sorafenib, an anti-proliferative and apoptogenic agent, is used as first-line treatment for glioblastoma. This study aimed to investigate the effect of sorafenib on TTFields-induced anti-tumor and anti-angiogenesis responses in glioblastoma cells in vitro and in vivo. Sorafenib sensitized glioblastoma cells to TTFields, as evident from significantly decreased post-TTFields cell viability (*p* < 0.05), and combinatorial treatment with sorafenib and TTFields accelerated apoptosis via reactive oxygen species (ROS) generation, as evident from Poly (ADP-ribose) polymerase (PARP) cleavage. Furthermore, use of sorafenib plus TTFields increased autophagy, as evident from LC3 upregulation and autophagic vacuole formation. Cell cycle markers accumulated, and cells underwent a G2/M arrest, with an increased G0/G1 cell ratio. In addition, the combinatorial treatment significantly inhibited tumor cell motility and invasiveness, and angiogenesis. Our results suggest that combination therapy with sorafenib and TTFields is slightly better than each individual therapy and could potentially be used to treat glioblastoma in clinic, which requires further studies.

## 1. Introduction

Glioblastoma, the most common primary brain tumor in adults, remains an incurable malignancy with a short expected survival [1]. For a long time, glioblastoma treatment included surgical cytoreduction followed by radiotherapy [2]. With this approach, the median survival was approximately 10 to 12 months [3,4]. In a recent study, co-administration of temozolomide (TMZ) with radiotherapy yielded better outcomes than radiotherapy alone [1]. With this new treatment standard, the expected median survival is 14.6 months, with a 2-year survival rate of 26.5% [5]. Tumor-treating fields (TTFields), alternating electric fields with a very low intensity (<2 V/cm) and an intermediate frequency (100–300 kHz), disrupt mitotic spindle formation during metaphase and effectively inhibit the growth of various human tumor cell lines, and have therefore been proposed to be useful in cancer treatment [6]. Accurate alignment of tubulin and septin is required for the initiation of cell division; this may be disrupted by TTFields [7]. Consequently, cancer cells in a G2/M arrest can be eliminated. Thus, TTFields have more pronounced effects on rapidly dividing cancer cells than on normal cells, implying that cancer cells can be selectively damaged. Another hypothesis regarding the mechanism underlying TTFields is the occurrence of chromosomal anomalies due to the inhibition of spindle formation by TTFields [8]. Chromosomal aberrations such as missegregation resulting from cell division can lead to apoptosis [9]. Furthermore, TTFields reportedly inhibit the localization of the septin complex, thereby disrupting cell division [7,10]. Recent clinical studies have reported that treatment of recurrent glioblastoma patients with TTFields may lead to longer overall survival than that observed with standard treatment, with no unexpected side effects [11]. In contrast, a randomized clinical trial reported that the outcomes for recurrent glioblastoma patients administered TTFields were not significantly better than those for patients administered conventional therapy [12]. The use of TTFields in combination with chemotherapeutic drugs increased the survival rate, without an increase in toxicity, compared with chemotherapy lone in a recent randomized clinical trial in newly diagnosed glioblastoma patients [13]. Previous studies have suggested that, although the clinical effectiveness of TTFields alone remains controversial, combinatorial therapy with TTFields and chemotherapy or radiotherapy are more efficient than monotherapy for newly diagnosed glioblastoma patients [14].

One potential chemotherapeutic agent for glioblastoma, sorafenib, is an oral multikinase inhibitor that blocks tumor cell proliferation and angiogenesis and induces tumor cell apoptosis by inhibiting serine/threonine kinases (c-RAF, and mutant and wild-type BRAF) and receptor tyrosine kinases, such as vascular endothelial growth factor receptors 2 and 3 (VEGFR2 and VEGFR3), platelet-derived growth factor receptor β (PDGFRβ), FLT3, and c-KIT [15]. In addition, sorafenib inhibits the mitogen activated protein kinase (MAPK)/extracellular-signal-regulated kinase (ERK) pathway, which plays an important role in cancer cell development [16,17], and inhibits eIF4E phosphorylation and downregulates Mcl-1 [17]. Sorafenib reportedly induces autophagy via LC3 upregulation, which occurs during autophagy and autophagy-related processes, including autophagic cell death [18,19,20]. Evaluation of sorafenib in phase I and II clinical trials on several forms of advanced solid tumors revealed favorable tolerability and promising clinical antitumor activity in advanced renal cell carcinoma, hepatocellular carcinoma, thyroid cancer, and osteosarcoma [21,22,23,24,25,26]. Moreover, clinical studies have used sorafenib in combination with various anticancer agents to treat several solid tumors [22]. The treatment efficacy of sorafenib with radiotherapy and temozolomide in glioblastoma patients has been investigated [27]. A desirable activity profile, preclinical evidence of antitumor activity in human malignant glioma models, and a promising safety profile have paved the way for recent phase I/II clinical trials in patients with malignant gliomas. Although phase I/II clinical trials have been conducted with sorafenib in combination with drugs such as temozolomide, bevacizumab, and temsirolimus, the therapeutic efficacy has not improved significantly [2,28,29]. Thus, sorafenib, a molecular targeting agent, and TTFields, a novel treatment modality, can be promising therapeutics that disrupt molecular defects in signaling pathways and may provide clinical benefits in treating glioblastomas.

This study aimed to investigate the mechanisms underlying the enhancement of TTFields-induced antitumor and anti-angiogenesis effects of sorafenib on glioblastoma. Our study provides a scientific rationale to evaluate this combinatorial strategy in clinical trials for TTFields therapy.

## 2. Results

### 2.1. Cooperative Effect of TTFields and Sorafenib on Glioblastoma Cancer Cell Proliferation

**Figure 1:**
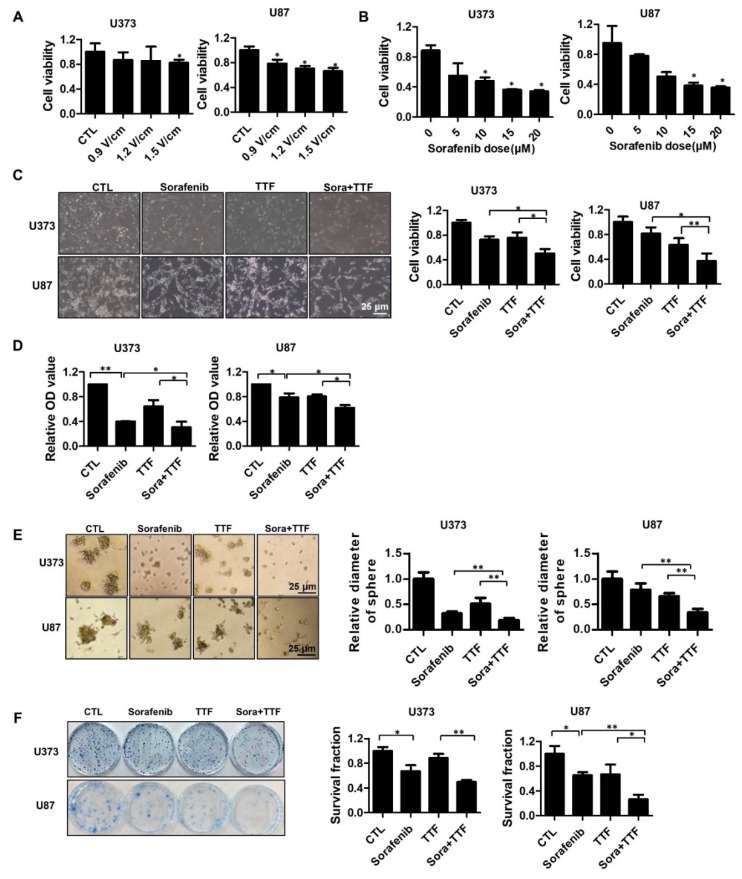
Tumor-treating field (TTField)-sensitizing effects of sorafenib on in vitro models of glioblastoma. (**A**) TTFields inhibited glioblastoma cell viability in an intensity-dependent manner. Cell viability was evaluated by cell counting using 0.4% Trypan Blue stain for U373 and U87 cells treated with TTFields for the indicated durations; * *p* < 0.05; (**B**) sorafenib inhibited glioblastoma cell Fluorine-18viability in a dose-dependent manner. Cell viability was evaluated by cell counting using 0.4% Trypan Blue stain for U373 and U87 cells treated with the indicated doses of sorafenib; * *p* < 0.05. (**C**–**E**) the viability of cells treated with a combination of TTFields and sorafenib was significantly lower than that of cells treated with either sorafenib or TTFields. The proliferation rate was detected by counting (**C**), MTT assay (**D**), and 3D colony culture (**E**). * *p* < 0.05; ** *p* < 0.01; (**F**) the sensitivity of U373 and U87 cells treated with sorafenib and TTFields was measured via a colony formation assay. The survival fraction, which was expressed as a function of the irradiation dose, was calculated as follows: survival fraction = colonies counted/(cells seeded × plating efficiency/100). * *p* < 0.05; ** *p* < 0.01. CTL: Control group; TTF: Tumor treating fields group.

To determine the optimal TTFields voltage, we subjected U373 and U87 cells to various voltages for 48 h (Figure 1A). The two glioblastoma cell lines exhibited a voltage-dependent reduction in cell viability (approximately 20% at 0.9 V/cm). To evaluate the effect of sorafenib on glioblastoma cells by the MTT (3-[4,5-dimethylthiazol-2-yl]-2,5 diphenyl tetrazolium bromide) assay, U373 and U87 cells were treated with sorafenib at different concentrations (Figure 1B). After 48 h, cell growth was significantly inhibited in cells treated with ≥5 µg/mL sorafenib (*p* < 0.05). These data indicated that U373 and U87 cells display dose-dependent sensitivity to sorafenib. In addition, the combination of sorafenib and TTFields treatment had a significantly greater antitumor effect on the U373 and U87 cells than either treatment alone, as evident from Trypan Blue and MTT cell viability assays (Figure 1C,D). Additionally, the colonies formed by mono-treated 3D cultures were larger than those formed upon combinatorial treatment (Figure 1E). In a colony formation assay, the surviving fractions decreased further in cells treated with TTFields plus sorafenib than in cells administered either of these treatments (Figure 1F). These data indicated that sorafenib has a TTFields-sensitizing effect on glioblastoma cells in vitro.

### 2.2. Sorafenib Promotes TTFields Sensitivity In Vivo

**Figure 2:**
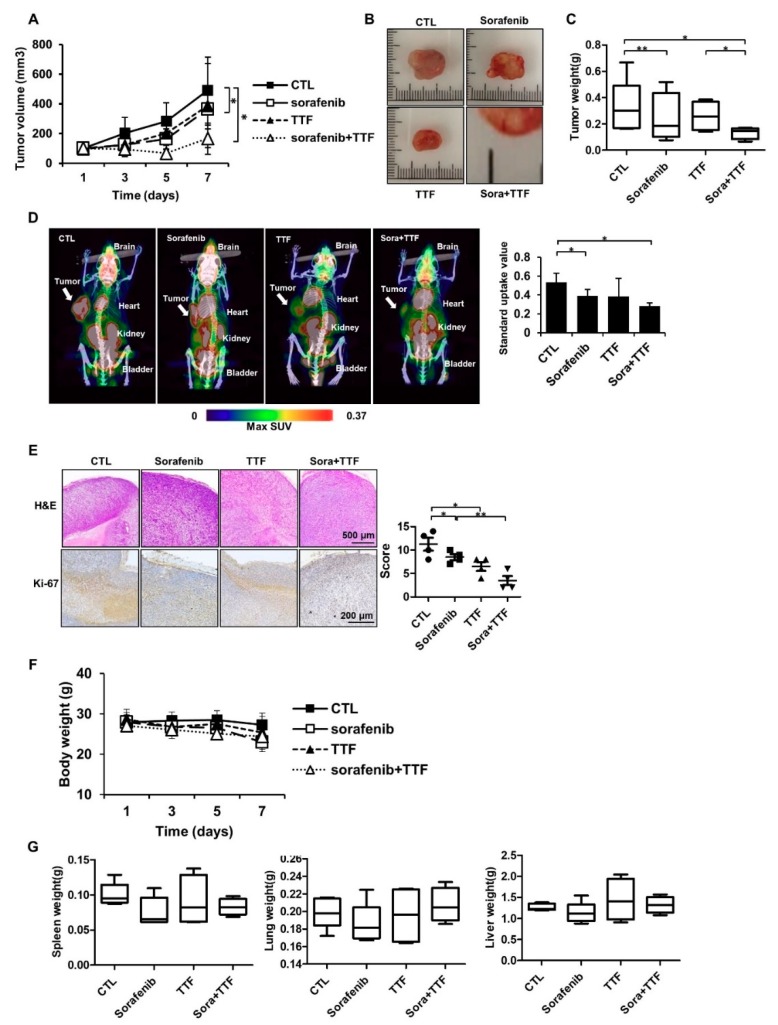
Tumor-treating field (TTField)-sensitizing effects of sorafenib on glioblastoma in vivo. (**A**) Nude mice were inoculated with U373 cells and treated with TTFields, sorafenib, or a combination thereof. Tumor volumes were measured at the indicated time points, using the formula: volume = (length × width^2^ × 3.14)/6 (*n* = 8); * *p* < 0.05; (**B**) images of tumors isolated from control- or TTFields-treated mice, *n* = 4, Sora: sorafenib.; bar = 1 cm (**C**) tumors were excised and weighed at the end of the experiment (seven days). * *p* < 0.05; ** *p* < 0.01; (**D**) representative PET/CT images of U373 tumor-bearing mice after injection of [^18^F]-fluorodeoxyglucose (FDG). The radioactivity of [^18^F]-FDG in tumors is presented as the maximum standard uptake value (mean ± SD). * *p* < 0.05; SUV: Standard uptake value. (**E**) hematoxylin and eosin (H&E) staining and Ki-67 expression was examined by immunohistochemistry. * *p* < 0.05; ** *p* < 0.01, *n* = 4; Solid circle: Control; Solid square: Sorafenib; Triangle: Tumor treating fields; Inverted triangle: Sorafenib+TTF. (**F**) the body weights of the mice were not significantly different among the sorafenib-, TTFields-, and combination-treated groups, *n* = 4; (**G**) the spleen, liver, and lung tissues of the mice were excised and weighed at the end of the experiment (seven days), *n* = 4.

To assess the effect of TTFields combined with sorafenib on glioblastoma growth in vivo, we used a subcutaneous glioblastoma model generated by injecting human U373 cells into mice. As shown in Figure 2A, xenografts treated with a combination of TTFields and sorafenib displayed decelerated growth compared to the control group and the groups receiving either of the treatments. Thus, tumors in the mono-treated groups were significantly larger than those in the group receiving combinatorial treatment (Figure 2B). Concurrently, tumor weight was reduced in the mice receiving combinatorial treatment compared to that in mice receiving either of the treatments (Figure 2C). As shown in Figure 2D, low uptake of [Fluorine-18(18F)]-fluorodeoxyglucose (FDG) was observed in tumors treated with TTFields plus sorafenib as compared to tumors receiving either of the treatments. The maximum standard uptake value was 0.53 ± 0.09 in the control group, 0.39 ± 0.07 in the sorafenib-treated group, 0.38 ± 0.19 in the TTFields-treated group, and 0.28 ± 0.03 in the combination-treated group (Figure 2D). Xenografts of mice receiving either of the treatments showed more intense Ki67 staining than those of mice receiving combinatorial treatment (Figure 2E). There were no visible signs of toxicity from TTFields or sorafenib administration in the mice, as evident from the absence of differences in body weight and the weights of various organs, including the spleen, lungs, and liver (Figure 2F,G). Together, these data suggested that TTFields combined with sorafenib inhibits the growth of glioblastoma in vivo.

### 2.3. Sorafenib Enhances TTFields-Induced Apoptosis

**Figure 3:**
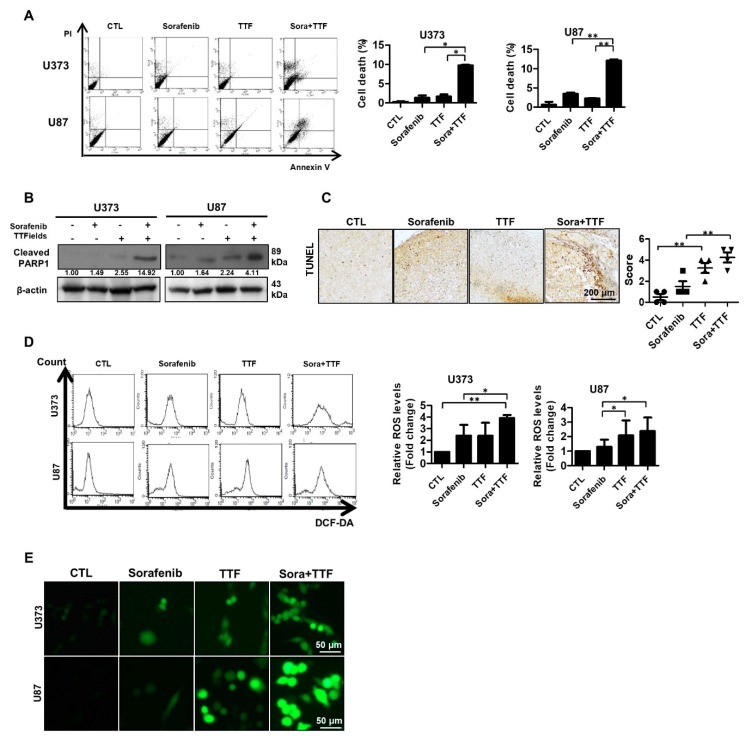
Effects of sorafenib and tumor-treating fields (TTFields) on apoptosis in glioblastoma cells. (**A**) U373 and U87 cells were exposed to sorafenib (5 µmol/L) and/or TTFields for 48 h prior to annexin V/PI staining; (**B**) cell lysates (30 µg) were immunoblotted with antibodies against cleaved PARP1 and β-actin; Band intensities were quantified and normalized to actin intensities (*n* = 3, mean ± SD). (**C**) terminal deoxynucleotide transferase-mediated dUTP nick-end labeling assays were performed using xenografts, *n* = 4; Solid circle: Control; Solid square: Sorafenib; Triangle: Tumor treating fields; Inverted triangle: Sorafenib+TTF. (**D**,**E**) U373 and U87 cells were treated with sorafenib, TTFields, or the indicated combinations, and reactive oxygen species (ROS) levels were determined using 2′,7′-dichlorofluorescein diacetate (a peroxide-sensitive dye), flow cytometry, and confocal laser fluorescence microscopy. Data are expressed as % of control and are means ± SD from 3 experiments. * *p* < 0.05; ** *p* < 0.01.

To investigate whether sorafenib and TTFields induce apoptosis, we assessed early apoptosis via annexin V and propidium iodide staining. In the two glioblastoma cancer cell lines, 48-h exposure to sorafenib plus TTFields significantly increased the proportion of early apoptotic cells (Figure 3A). Thereafter, we investigated whether sorafenib-enhanced TTFields cytotoxicity resulted from increased PARP cleavage, leading to enhanced apoptotic cell death. We observed increased PARP cleavage in response to combined TTFields and sorafenib treatment when compared to treatment with sorafenib alone (Figure 3B). To determine whether combinatorial therapy induces apoptosis in vivo, we evaluated the apoptotic rate using a terminal deoxynucleotidyl transferase-mediated dUTP nick-end labeling (TUNEL) assay. Apoptotic cell death was increased upon combinatorial treatment (Figure 3C). We also investigated the association between ROS production and enhancement of TTFields-induced apoptosis by sorafenib. ROS production was more strongly induced upon combinatorial treatment than upon individual treatments (Figure 3D), which may explain the increased the apoptotic rate upon combinatorial treatment. These data are consistent with the results of fluorescence microscopy, as shown in Figure 3E.

### 2.4. Effects of Sorafenib and TTFields on Autophagic Cell Death

**Figure 4:**
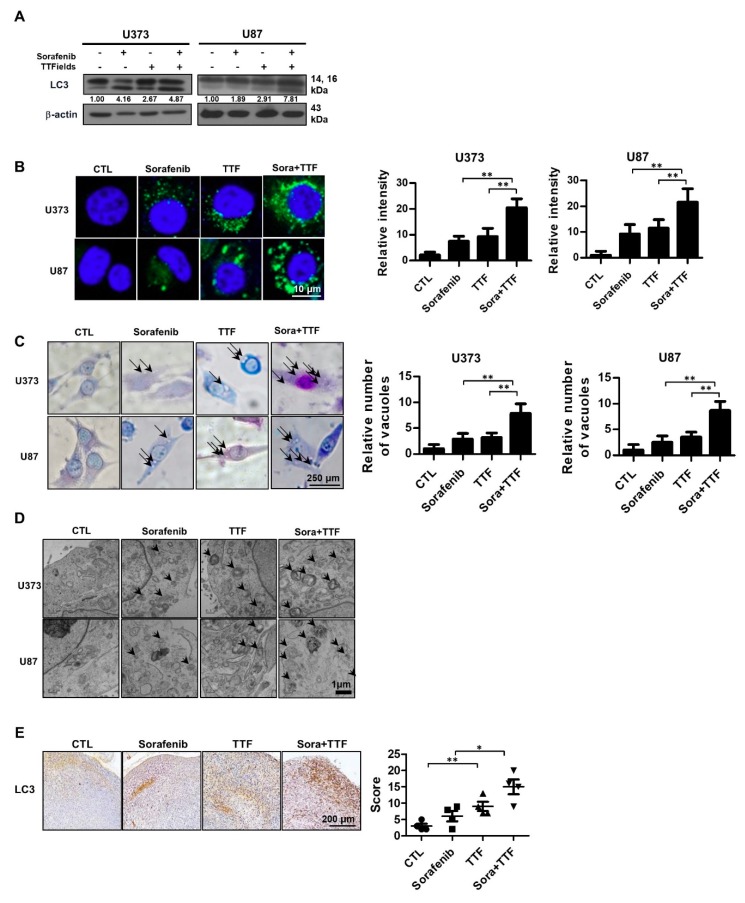
Combinatorial treatment with sorafenib and tumor-treating fields (TTFields) induces autophagy in glioblastoma cancer cells. (**A**) cell lysates (30 µg) were immunoblotted with anti-LC3 and anti-β-actin antibodies; Band intensities were quantified and normalized to actin intensities (*n* = 3, mean ± SD). (**B**) cyto-ID staining of U373 and U87 cells with and without sorafenib or with and without TTFields treatment. ** *p* < 0.01; (**C**) cells were stained with Giemsa stain (10% in phosphate-buffered saline), washed, and imaged using a Leica DM IRB light microscope (magnification, 40×). Black arrows indicate vacuoles. ** *p* < 0.01; (**D**) autophagy was assessed by transmission electron microscopy in cells, bar = 1 µm; black arrow: autophagic vacuoles. (**E**) LC3 expression in xenografts was examined by immunohistochemistry. Representative images are presented. * *p* < 0.05; ** *p* < 0.01; *n* = 4; Solid circle: Control; Solid square: Sorafenib; Triangle: Tumor treating fields; Inverted triangle: Sorafenib+TTF.

To investigate the anticancer effects of sorafenib and TTFields further, we examined other cellular responses associated with cell death upon sorafenib or TTFields treatment; in particular, we investigated the effects on autophagy, since both TTFields and sorafenib induce autophagy [20,30]. Western blotting revealed that the levels of LC3, a specific marker of autophagosome generation, were increased in cells administered combinatorial treatment (Figure 4A). As shown in Figure 4B, increased accumulation of Cyto-ID Green, an autophagy indicator, was observed around combination-treated U373 and U87 cells. After 48 h of treatment with TTFields plus sorafenib, Giemsa-stained U373 and U87 cells exhibited ultrastructural changes in the whole cytoplasm and membranes, including loss of plasma membrane integrity and distinct vacuole formation, compared to those receiving individual treatments (Figure 4C). This drastic vacuolization of the cytoplasm without apparent loss of nuclear material is consistent with the described macrostructure of autophagosomes. In addition, transmission electron microscopy was used to verify autophagosome formation in cells receiving combinatorial treatment. As shown in Figure 4D, cells administered combinatorial treatment exhibited accumulation of large autophagic vacuoles with a typical double-layer membrane and organelle remnants, whereas only a few vacuoles were observed in cells receiving individual treatments. In addition, mouse xenografts were stained for LC3 to clarify whether sorafenib combined with TTFields could additionally induce autophagy in vivo compared to the individual treatments (Figure 4E). Collectively, our data showed that autophagy contributes to glioblastoma cell death after combinatorial treatment in vitro and in vivo.

### 2.5. Effects of Sorafenib and TTFields on the Cell Cycle

**Figure 5:**
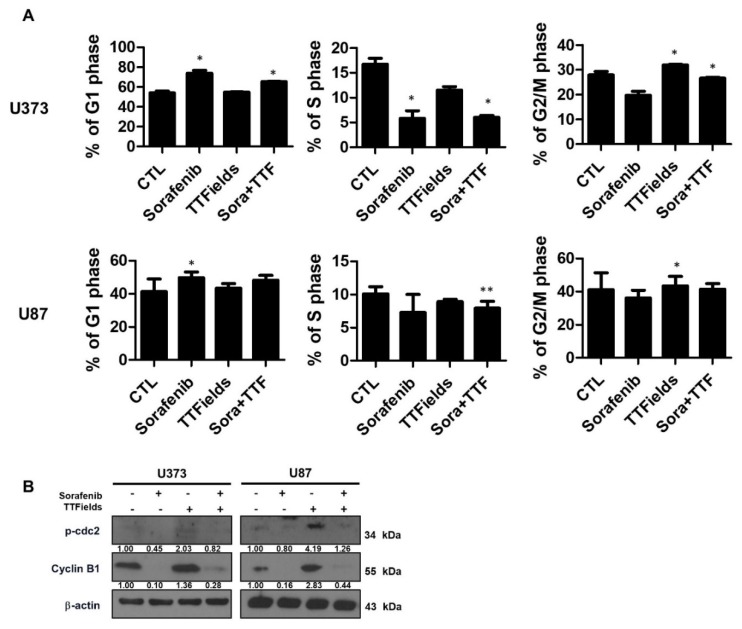
Sorafenib plus tumor-treating fields (TTFields) inhibits cell cycle progression in glioblastoma cells. (**A**) U373 and U87 cells were treated with sorafenib (5 µmol/L) and/or 0.9 V/cm TTFields for 24 h. Cell cycle distribution was analyzed quantitatively by flow cytometry. * *p* < 0.05; ** *p* < 0.01; (**B**) phospho-cdc2 and cyclin B1 expression was analyzed by Western blotting. β-Actin served as a loading control. Equal amounts of cell lysate (30 µg) were electrophoresed and analyzed; Band intensities were quantified and normalized to actin intensities (*n* = 3, mean ± SD).

We analyzed cells treated with 5 μM sorafenib or TTFields for 24 h by using propidium iodide staining and flow cytometry to evaluate the effect of sorafenib treatment on cell cycle progression in human glioblastoma cells. Sorafenib treatment increased the proportion of cells in the G1 phase and markedly decreased the proportion of cells in the S phase in comparison with the control treatment (Figure 5A). Similar results were obtained when using U87 cells. When the U373 and U87 cells were exposed to TTFields alone for 24 h, a small fraction of the cells underwent G2/M arrest and the percentages of cells in the G1 and S phases also decreased. Upon pretreatment with sorafenib, the TTFields-induced G2/M phase arrest decreased within 24 h after TTFields exposure, with an increase in the percentage of cells in the G1 phase compared with that observed with TTFields alone. Western blotting indicated that TTFields alone led to significant accumulation of cyclin B and p-CDC2, which are key regulators of the G2/M transition (Figure 5B). Combinatorial treatment with sorafenib suppressed the TTFields-induced accumulation of these markers.

### 2.6. Combinatorial Treatment Significantly Inhibits Tumor Cell Motility and Invasion, and Angiogenesis

**Figure 6:**
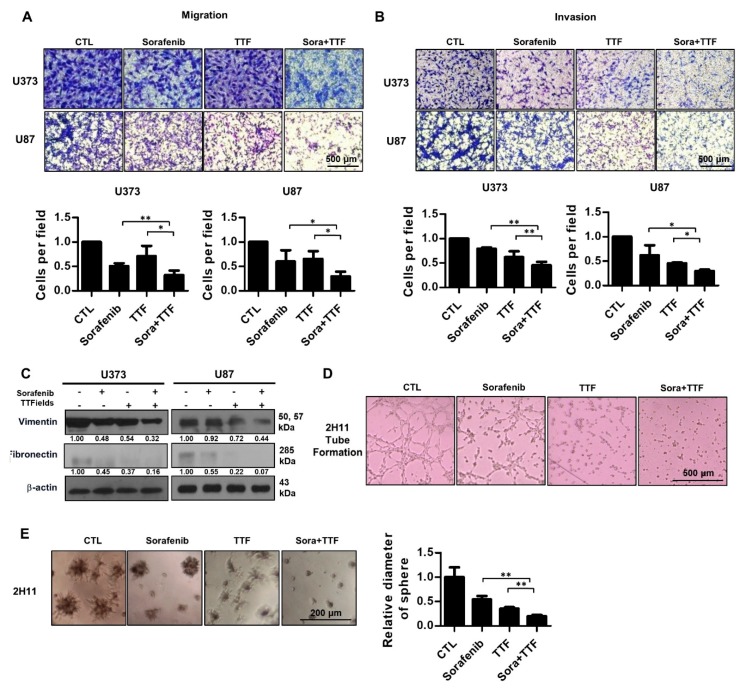
Effect of combinatorial treatment with Sorafenib and tumor-treating fields (TTFields) on the invasiveness and migration of glioblastoma cells. (**A**) tumor cell migration was assessed using a Transwell chamber assay. * *p* < 0.05; ** *p* < 0.01, bar = 500 µm; (**B**) tumor cell invasion was assessed using a Matrigel invasion assay. * *p* < 0.05; ** *p* < 0.01, bar = 500 µm; (**C**) cell lysates prepared from sorafenib-, TTFields-, and sorafenib plus TTFields-treated cells were used in Western blotting using antibodies against vimentin and fibronectin; Band intensities were quantified and normalized to actin intensities (*n* = 3, mean ± SD). (**D**) tube formation assay using 2H11 cells subjected to the indicated treatments; (**E**) 3D colony cultures of 2H11 cells treated as indicated. ** *p* < 0.01.

To determine whether sorafenib regulates the effects of TTFields on metastasis, we examined the effects of sorafenib and TTFields on U373 and U87 cell migration. A Transwell chamber assay revealed that combinatorial treatment decreased cell migration in the U373 and U87 cell lines after 24 h (Figure 6A). Furthermore, using a Matrigel invasion assay, we investigated whether sorafenib and TTFields could affect the invasiveness of glioblastoma cells. Serum-starved cells were seeded in the upper chambers of the Transwell assay system, and the cells that penetrated the Matrigel barrier in response to a chemoattractant (serum) were counted at various time points. Combinatorial treatment significantly decreased the number of invading U373 and U87 cells as compared to the individual treatments (Figure 6B). Together, these results suggested that sorafenib plus TTFields might effectively inhibit the migration and invasiveness of human glioblastoma cells. To investigate the molecular mechanism underlying the modulation of the expression of epithelial-to-mesenchymal transition markers by combinatorial treatment, Western blotting was used. Vimentin and fibronectin, mesenchymal markers, were downregulated in both cell lines (Figure 6C). Furthermore, we examined whether combinatorial treatment would block angiogenesis. Combinatorial treatment completely inhibited tube formation in 2H11 cells compared with the individual treatments (Figure 6D). In addition, colonies formed in control 3D 2H11 cell cultures were larger than colonies formed by cells treated with TTFields or sorafenib alone, whereas colonies formed by cells treated with the combination were the smallest (Figure 6E).

## 3. Discussion

This study aimed to investigate the mechanism underlying TTFields sensitization of glioblastoma cells by sorafenib to facilitate the clinical use of sorafenib as a TTFields sensitizer. The Food and Drug Administration (FDA) approved the use of TTFields for recurrent glioblastoma [31]. Recently, a phase III clinical study reported that the use of a combination of 200-kHz TTFields and adjuvant TMZ to treat newly diagnosed glioblastoma enhanced both progression-free and overall survival [31]. Based on this finding, the FDA recently approved the use of TTFields to treat newly diagnosed glioblastoma. While this treatment system is very advanced, to obtain improved clinical outcomes, clinically effective drugs need to be used with TTFields. Among anticancer drugs, we focused on sorafenib, a well-known multikinase inhibitor. The availability of drugs targeting novel cellular pathways has increased the possibility of developing improved treatments for glioblastoma patients. Studies on various angiogenesis inhibitors have highlighted the importance of angiogenesis in cancer growth and progression. Recently, several clinical trials have been initiated to evaluate the use of sorafenib in combination with various anticancer drugs to treat various tumors. The most promising evidence of antitumor activity was observed when sorafenib was combined with interferon-α in renal cell carcinoma, dacarbazine in melanoma, doxorubicin in hepatocellular carcinoma, and gemcitabine in ovarian cancer [24]. Moreover, the combination of sorafenib and another targeted agent, bevacizumab, yielded remarkable antitumor effects in ovarian cancer patients [25], and other clinical studies have reported that the combination of sorafenib and radiation might provide clinical benefits in treating various cancers, including glioblastoma [22,26,27]. However, the mechanism underlying TTFields-mediated enhancement appears somewhat more complex than predicted previously in studies on glioblastoma.

We aimed to provide a scientific rationale for the clinical application of sorafenib as a TTFields sensitizer in glioblastoma treatment. Our results suggest that sorafenib significantly enhances the therapeutic efficiency of TTFields through inhibition of tumor cell survival, apoptosis, cell cycle regulation, autophagy, inhibition of tumor cell invasiveness, and inhibition of angiogenesis in human glioblastoma cell lines. Combinatorial treatment with sorafenib and TTFields inhibited the proliferation of U373 and U87 cells in vitro (Figure 1). Moreover, in nude mice bearing xenografts of U373 glioblastoma cells, combinatorial treatment inhibited tumor growth and prolonged the survival of the animals (Figure 2). Notably, sorafenib at concentrations >5 μM induced significant cytotoxicity. Sorafenib-treated cells were more sensitive to TTFields than non-treated cells (Figure 1b). These results showed that sorafenib enhanced the sensitivity of U373 and U87 cells to TTFields. Combination with sorafenib significantly enhanced the sensitivity of glioblastoma cancer cells to TTFields by promoting apoptosis via increased ROS production (Figure 3). In addition, sorafenib increased autophagy induced by TTFields (Figure 4). Flow cytometry revealed that treatment with sorafenib, alone or in combination with TTFields, inhibited cell cycle progression (Figure 5). Administration of TTFields with sorafenib significantly decreased invasiveness and angiogenesis (Figure 6).

Despite this combinatorial effect, the critical persistent issue is that the treatment of glioblastoma is complicated by the blood–brain barrier (BBB), which is a physiological obstacle for drug delivery to the central nervous system. The effect of TTFields on the BBB is yet unclear. Various tools have been developed for local drug delivery to brain tumors, including convection-enhanced delivery [32]. Local delivery of sorafenib to malignant cells in the brain may increase the effectiveness of antitumor activity with reduced systemic toxicity. Sorafenib exhibited high tolerability and promising antitumor effects in clinical trials in various types of solid tumors [33,34,35]. Thus, sorafenib is a potentially promising drug to treat malignant gliomas in combination with TTFields. There is a need for optimizing clinical trials of electric field-based tumor treatments via preclinical testing using patient samples and the application of electric fields alone or in combination with drugs. Furthermore, an ideal TTFields sensitizer enhances the sensitivity of tumor cells to TTFields and is harmless to or protects normal tissue. It is unclear whether sorafenib protects normal tissues in combined treatment with TTFields. However, despite the numerous clinical trials of sorafenib for various solid tumors, interest in clinical trials on glioblastoma remains low. Patients with glioblastoma, however, have few therapeutic options, with most of these options being palliative. The data produced in this study suggest that the use of sorafenib and TTFields is a valid therapeutic option for treating glioblastoma that warrants further investigation. The efficacy of TTFields has been reported in various cancers, such as non-small cell lung cancer, pancreatic cancer, ovarian cancer, mesothelioma, liver cancer, and glioblastoma [10,36]. Sorafenib is also applicable for treating various cancers, such as renal cell carcinoma, hepatocellular carcinoma, and thyroid cancer [24,25,37]. Therefore, combinatorial treatment with sorafenib and TTFields may be effective for treating various cancers in clinical practice.

In line with our findings, Kessler et al. recently reported that combinatorial use of the spindle assembly checkpoint inhibitor MPS1-IN-3 and TTFields to target glioblastoma cells increased apoptosis as well as the rate of cell cycle arrest [38]. However, sorafenib is an anticancer agent that causes cell cycle arrest in the G1 phase. Therefore, unlike MPS1-IN-3, which increased G2/M phase when administered in combination with TTFields, sorafenib reduced it. In addition, both MPS1-IN-3 and sorafenib promoted apoptosis and suppressed proliferation, suggesting that TTFields may produce synergistic effects with various therapeutic agents.

In summary, we report that sorafenib can increase the sensitivity of glioblastoma cancer cells to TTFields through inhibition of tumor cell survival, apoptosis, cell cycle regulation, autophagy, inhibition of tumor cell invasiveness, and inhibition of angiogenesis. These findings provide a molecular basis for the use of chemotherapeutic drugs as TTFields sensitizers to treat cancer. In the future, the identification of TTFields seems to be key for the optimization of therapeutic strategies for glioblastoma.

## 4. Materials and Methods

### 4.1. Experimental Setup for Electric Fields

TTFields were generated with a pair of insulated wires (Seoul Electric Wire Co., Ltd., Chungcheongbuk-do, Republic of Korea; outer diameter, 0.4 mm; polyvinyl chloride insulation thickness, 0.17 mm; dielectric breakdown, 25 kV/mm) connected to a function generator (AFG-2112, Good Will Instrument Co., Ltd., Taiwan) and a high-voltage amplifier (A303, A. A. Lab Systems Ltd., Ramat Gan, Israel) that generated sine-wave signals of 0–800 V [39]. To apply the electric field to cells, the insulated wires were attached to the bottom of each cell dish, 1 cm from each other. The applied electric field intensity and frequency was 0.9–1.5 V/cm and 150 kHz, respectively, for all experiments. To confirm the voltage, the same culture dishes as those used in the in vitro experiments were separately prepared, and the voltages applied to the cells were measured using an oscilloscope (GDS-2102A, Good Will Instrument Co. Ltd., New Taipei, Taiwan), while considering the interference caused by the culture dishes [39]. We maintained the frequency at 150 kHz in this experiment because this reportedly is the optimum frequency for U373 glioblastoma cells (cell line used in in vitro experiments) [39].

### 4.2. Antibodies and Chemicals

Anti-cleaved PARP (#9541), anti-LC3 (#12741) anti-p-cdc2 (#4139), anti-cyclin B (#4138), anti-vimentin (#3932), and anti-β-actin (#3700) were purchased from Cell Signaling Technology (Danvers, MA, USA). Anti-fibronectin (ab2413) was purchased from Abcam (Cambridge, UK). Sorafenib tosylate (marketed as Nexavar by Bayer, Leverkusen, Germany) was purchased from Selleckchem (Houston, TX, US). For in vitro experiments, sorafenib was dissolved in dimethyl sulfoxide to generate a 20 mmol/L stock solution, which was stored at 4 °C until use.

### 4.3. Cell Culture

Human glioblastoma U87 and U373 cell lines were obtained from the Korean Cell Line Bank (Seoul, Korea). U87 and U373 cells were cultured in Dulbecco’s Modified Eagle’s Medium (DMEM) supplemented with 10% fetal bovine serum (FBS), glutamine, hydroxyethyl-piperazineethanesulfonic acid buffer (HEPES), and antibiotics in a humidified incubator at 37 °C under 5% CO_2_. The murine endothelial cell line 2H11 was cultured in DMEM supplemented with 10% FBS in a humidified 10% CO_2_ environment.

### 4.4. Cell Viability Assay

Cells were seeded at a density of 5000 cells/well in a 96-well plate and incubated for 24 h, in accordance with the indicated experimental conditions. To quantify cell viability, an equal volume of culture medium containing EZ-Cytox reagent (EZ3000, Daeillab Service, Chungcheongbuk-do, Republic of Korea) was added to the cells, and the mixture was incubated for 4 h. Cell viability was determined by measuring the absorbance at 450 nm using a Multiskan EX (Thermo Fisher Scientific; Waltham, MA, US).

### 4.5. 3D Culture System

Human glioblastoma U373, U87, and 2H11 cells were seeded in 96-well plates at a density of 1 × 10^4^ cells/well. The 96-well plates had been precoated with Matrigel as a basement membrane by adding 40 µl of Matrigel to each well, followed by incubation at 37 °C for 30 min. Cells were seeded onto the gel in appropriate medium, and the wells were photographed 10 days later.

### 4.6. Colony Formation Assay

Cells were subjected to TTFields 6 h after sorafenib exposure at a final concentration of 5 μmol/L, after which cells were incubated for 48 h. After 14–20 days, colonies were stained with 0.4% Crystal Violet (Sigma, St. Louis, MO, USA). The plating efficiency indicates the percentage of seeded cells of a particular cell line that formed colonies under specific culture conditions. The survival fraction, expressed as a function of irradiation, was calculated as follows: survival fraction = colonies counted/(cells seeded × plating efficiency/100).

### 4.7. Tumor Xenografts in Nude Mice

A single-cell suspension (2 × 10^6^ cells) was subcutaneously injected into the flanks of 5-week-old BALB/c nude mice (Nara Biotech; Gyeonggi-do, Republic of Korea). When the tumor reached a minimal volume of 100–200 mm^3^, 1 V/cm TTFields, 30 mg/kg sorafenib (three times a week) via intraperitoneal injections, or the combination were initiated and continued for 7 days. Tumor volumes were determined with the formula (L × *l*^2^)/2 by measuring tumor length (L) and width (*l*) with a caliper. The sample size was determined to be 5 per group because this number of mice was required to achieve an effect size of 0.85, a significance level of ≤5%, and a power of ≥80% in Student’s *t*-test. Mice that desorbed from the electrodes during TTFields treatment were excluded. There was no randomization when animals were allocated to experimental groups. The trial was approved by Korea University Institutional Review Board (KUIACUC-2018-73, 1 June 2018).

### 4.8. Positron Emission Tomography (PET)/Computed Tomography (CT) Acquisition

A Siemens Inveon PET scanner (Siemens Medical Solutions, Erlangen, Germany) was used for PET imaging [40]. Before ^18^F-fluoro-2-deoxy-d-glucose ([^18^F]-FDG) uptake, the mice were warmed using a heating pad. Thereafter, 200 μCi of [^18^F]-FDG was injected into the tail vein, and the mice were anesthetized with 2% isoflurane in 100% oxygen (Forane solution, ChoongWae Pharma, Seoul, Korea). To acquire anatomical images, X-ray CT data for the mice were acquired with full rotation and 180 projection, using the Inveon system. The exposure time was 200 ms, and the estimated scan time was 504 s for X-ray CT. X-ray CT data were reconstructed using Feldkamp reconstruction (L.A. Feldkamp et al., Dearborn, MI, US) with Shepp and Logan filters. The effective pixel size of the reconstructed X-ray CT image was 109.69 μm × 109.69 μm. Thirty minutes after tracer uptake and acquisition of X-ray CT data, PET data were acquired for 15 min within an energy window of 350–650 keV. The emission list-mode PET data were sorted into 3D sinograms and reconstructed using OSEM2D methods. The pixel size of the reconstructed images was 0.38 × 0.38 × 0.79 mm^3^. All relevant corrections, such as normalization, dead-time correction, and random correction, were performed for all datasets. X-ray CT data were used to delineate the region of interest (ROI). PET and CT images were coregistered using Inveon Research Workplace (version 2.0, Erlangen, Germany) (Siemens Medical Solutions). After coregistration of the CT and PET data, the ROI was delineated on the CT image and included in the PET data. The maximum pixel values within the ROI on the PET image were then measured and converted to radioactivity cpm values, using a predetermined conversion factor. The standard uptake value was determined by measuring the tissue concentration (MBq/mL)/injected dose (MBq)/body weight (g).

### 4.9. Detection of Apoptotic Cells via Annexin V Staining

After sorafenib exposure, cells were subjected to TTFields and then incubated for an additional 48 h. The cells were washed with ice-cold phosphate-buffered saline (PBS), trypsinized, and resuspended in 1× binding buffer (10 mM HEPES/NaOH [pH 7.4], 140 mM NaCl, and 2.5 mM CaCl_2_) to obtain a cell density of 1 × 10^6^ cells/mL. Aliquots (100 μL) of the cell solution were mixed with 5 μL of annexin V conjugated with fluorescein isothiocyanate (PharMingen; San Jose, CA, US) and 10 μL of a propidium iodide stock solution (50 μg/mL in PBS) by gentle vortexing, followed by 15 min of incubation at room temperature in the dark. Buffer (400 μL, 1×) was added to each sample, and the samples were analyzed on a FACScan flow cytometer (Becton Dickinson, Franklin Lakes, NJ, USA). A minimum of 10,000 cells were counted for each sample, and data were analyzed using CellQuest software (version 6.0, BD Biosciences, San Jose, CA, US).

### 4.10. Western Blotting

After sorafenib treatment, glioblastoma cells were subjected to TTFields and then incubated for 24 or 48 h. Then, the cells were lysed with Radioimmunoprecipitation (RIPA) buffer; proteins were separated by sodium dodecyl sulfate-polyacrylamide gel electrophoresis and electro-transferred to nitrocellulose membranes. The membranes were blocked with 1% (*v*/*v*) nonfat dried milk in Tris-buffered saline with 0.05% Tween-20 and incubated with the appropriate antibodies. Primary antibodies were used at a 1:1000 dilution; secondary antibodies, 1:5000. Immunoreactive protein bands were visualized via enhanced chemiluminescence (Amersham Biosciences; Little Chalfont, UK) and scanned.

### 4.11. TUNEL Assay

Tumors were harvested and fixed with 10% neutral-buffered formalin. Deparaffinized sections were incubated with 20 μg/mL protease K for 15 min at room temperature, washed with PBS, and incubated with the TUNEL reaction mixture (Millipore, Burlington, MA, USA) for 1 h at 37 °C in a humidified chamber. The analysis was carried out in a blinded manner.

### 4.12. Fluorescence-Based Quantification of Intracellular ROS

The fluorescent probe 2’,7’-dichlorofluorescin diacetate (DCFH-DA) was used to quantify intracellular ROS. For fluorocytometric analysis, U373 and U87 cells were treated for 48 h with TTFields, sorafenib, or a combination and were loaded with 10 μM DCFH-DA in 5 mL of PBS min at room temperature for 30. Fluorescence was measured using a flow cytometer (Becton Dickinson, Franklin Lakes, NJ, USA). A minimum of 10,000 cells were counted for each sample, and data were analyzed using CellQuest software (BD Biosciences, San Jose, CA, US). In addition, the DCFH-DA-loaded cells were observed under a confocal laser scanning microscope (LSM 880, ZEISS, Oberkochen, Germany).

### 4.13. Autophagy Assay

Cells were treated, harvested, stained with Cyto-ID^®^ Green detection reagent (Cyto-ID^®^ Autophagy Detection Kit 2.0, Enzo Life Science, Farmingdale, NY, US) and Hoechst 33342 in accordance with the manufacturer’s protocols [41], and observed under a confocal laser scanning microscope (LSM 880).

### 4.14. Giemsa Staining

U373 and U87 cells were treated for 48 h with TTFields, sorafenib, or a combination of both, and Giemsa staining was performed using a kit from Sigma (St. Louis, MO, US) (GS500). Briefly, cells (10^4^ cells/well) were seeded in six-well plates and were allowed to adhere overnight on cover slips, followed by treatment with TTFields, sorafenib, or a combination of both. The cells were fixed with 4% paraformaldehyde for 10 min and stained with Giemsa (10% in PBS) for 15 min, followed by washing with tap water. Images were acquired using a Nikon Eclipse Ts2R-FL (Tokyo, Japan).

### 4.15. Transmission Electron Microscopy

U373 and U87 cells were treated for 48 h with TTFields, sorafenib, or a combination of both and then fixed in 2.5% glutaraldehyde (Sigma; St. Louis, MO, US). A Sorvall MT5000 microtome (DuPont Instruments, MT5000, Columbus, OH, US) was used to prepare ultrathin sections after dehydration. The sections were stained with lead citrate and/or 1% uranyl acetate, and autophagic vacuoles in the cytoplasmic area were quantified using Image Pro Plus software (version 3, Rockville, MD, US).

### 4.16. Immunohistochemistry

For immunohistochemical analysis, 4-μm-thick paraffin-embedded glioblastoma sections were mounted on coated glass slides to detect proteins of interest. Following antigen retrieval and blocking of endogenous peroxidases and nonspecific protein binding, slide sections were first incubated with primary antibodies (anti-Ki67 and anti-LC3 [1:200]; Cell Signaling Technology, Danvers, MA, USA), followed by incubation with horseradish peroxidase-conjugated secondary antibodies. Slides were developed with 3,3’-diaminobenzidine, followed by hematoxylin counterstaining. The analysis was carried out in a blinded manner.

### 4.17. Flow Cytometry

Cells were cultured, harvested at the indicated times, and stained with propidium iodide (1 μg/mL, Sigma, St. Louis, MO, US) in accordance with the manufacturer’s protocol. Then, the cells were analyzed on a FACScan flow cytometer (Becton Dickinson, Franklin Lakes, NJ, USA). A minimum of 10,000 cells were counted for each sample, and data were analyzed using CellQuest software (version 6.0, BD Biosciences; San Jose, CA, US).

### 4.18. Invasion/Migration Assay

Invasiveness was measured in vitro using Transwell chambers, in accordance with the manufacturer’s protocol. Briefly, cells were seeded onto the membrane of the upper chamber of the Transwell at a concentration of 4 × 10^5^ cells/mL in 150 μL of DMEM and were left untreated or were treated with the indicated doses of sorafenib, TTFields, or a combination of both for 48 h. The medium in the upper chamber was serum-free, whereas that in the lower chamber contained 10% FBS as a source of chemoattractants. Cells that penetrated the Matrigel or gelatin-coated membrane were stained with cell stain solution containing crystal violet, supplied with the Transwell chamber assay (Chemicon, Millipore, Billareca, MA, USA), and were photographed after 48 h of incubation.

### 4.19. Matrigel-Based In Vitro Endothelial Tube Formation Assay

Endothelial cell tube formation was assessed using Matrigel-coated chamber slides, as described previously [42]. The results of each assay were photographed (Nikon Eclipse Ti microscope with a DS-Fi1 camera, Tokyo, Japan) at a magnification of 40×, and the total area occupied by endothelial cell-derived tubes in each chamber was calculated using NIS-Elements-Basic Research software (version number) (Nikon; Tokyo, Japan) and was expressed as an angiogenic score.

### 4.20. Statistical Analysis

Means were compared using Student’s *t*-test. Differences were considered significant if the *p*-value was less than 0.05 or 0.01.
